# Enteral Cilostazol and High-Dose Intravenous Albumin in Aneurysmal Subarachnoid Hemorrhage Patients With Refractory Cerebral Ischemia

**DOI:** 10.7759/cureus.71566

**Published:** 2024-10-15

**Authors:** Navpreet K Bains, Minh Ngo, Ibrahim A Bhatti, Francisco E Gomez, Niraj A Arora, Premkumar N Chandrasekaran, Farhan Siddiq, Camilo R Gomez, Jose I Suarez, Adnan I Qureshi

**Affiliations:** 1 Neurology, Zeenat Qureshi Stroke Institute, St. Cloud, USA; 2 Neurology, University of Missouri, Columbia, USA; 3 Neurological Surgery, University of Missouri, Columbia, USA; 4 Neurology, Johns Hopkins Medicine, Baltimore, USA

**Keywords:** albumin, aneurysmal subarachnoid hemorrhage, cerebral vasospasm, cilostazol, refractory cerebral ischemia

## Abstract

Cerebral ischemia associated with vasospasm in patients with aneurysmal subarachnoid hemorrhage (aSAH) requires a multifaceted approach. We report the use of the combination of enteral cilostazol and intravenous (IV) high-dose albumin in aSAH patients with cerebral ischemia refractory to other accepted pharmacologic and endovascular treatments. Three aSAH patients who developed cerebral ischemic symptoms despite treatment with oral nimodipine and endovascular measures (i.e., intraarterial vasodilators and balloon angioplasty) were treated with enteral cilostazol (200 mg/day) and one or more doses of IV (25%) albumin (1.25 g per kg over eight hours). The patients were monitored by serial neurological examinations, transcranial Doppler imaging (TCDI) ultrasound, computed tomographic angiography (CTA), and perfusion (CTP) scans.

Three patients (ages 58, 67, and 56 years) developed symptomatic cerebral ischemia and vasospasm following an aSAH. Due to limited angiographic response to endovascular treatment, including intraarterial vasodilators with or without balloon angioplasty, IV (25%) albumin and enteral cilostazol were administered. CT angiogram and perfusion 2-3 days post-treatment demonstrated resolution of the perfusion deficits and angiographic vasospasm. Concurrently, TCDI demonstrated improved vasospasm and clinical examination demonstrated resolution of neurological deficits. None of the patients required any additional treatments for cerebral ischemia. A combination of oral cilostazol and IV high dose (25%) albumin was associated with amelioration of angiographic vasospasm, reduction of tissue perfusion deficits, and clinical improvement of aSAH patients with severe refractory cerebral ischemia.

## Introduction

Cerebral ischemia associated with cerebral vasospasm in patients with aneurysmal subarachnoid hemorrhage (aSAH) is a multifactorial process that includes smooth muscle and fibroblast proliferation, microembolism, endothelial injury, and a tissue catabolism state [1]. Vasospasm involving medium-sized intracranial arteries remains a major cause of cerebral ischemia in patients with aSAH [2]. Cerebral ischemia is also attributed to other processes, such as microembolism and small vessel vasospasm [3]. Therefore, aSAH-associated cerebral ischemia, especially when refractory to conventional therapeutic measures, may require a multifaceted approach [4]. Enteral cilostazol and high-dose intravenous (IV) albumin have been associated with reduced angiographic vasospasm, cerebral ischemia, and death or disability in several clinical trials [5-8]. Cilostazol, a phosphodiesterase III inhibitor, increases the activation of intracellular cAMP, inhibits vascular smooth muscle proliferation and platelet-derived growth factor production, and increases the release of nitric oxide from endothelial cells, thereby exerting vasodilatory and anti-inflammatory effects [8]. Albumin, a protein synthesized by the hepatocytes, composes 50-60% of blood plasma proteins and 80% of the colloidal osmotic pressure in the blood, thereby improving microcirculatory blood flow by increasing serum oncotic pressure [9]. Albumin also decreases leukocyte rolling and adherence, thus reducing inflammatory response and protecting the integrity of the blood-brain barrier [7,10]. We report the use of a combination of both of these agents in aSAH patients who displayed symptomatic cerebral ischemia refractory to conventional treatment measures.

## Case presentation

Three patients admitted to University Hospital, Columbia, Missouri, with aSAH were included in this report. On admission, they all underwent baseline computed tomography (CT) studies, including brain CT and angiography (CTA) scans. A modified Fisher score was assigned based on this initial brain CT [5]. In addition, we assigned a Hunt and Hess score to specify the severity of the SAH in each patient [11,12]. All three patients underwent aneurysm securement by various methods within 24 hours of their admission and were monitored in a dedicated neurointensive care unit (NICU); one patient underwent surgical clipping, while the other patients underwent endovascular treatment. Prior to aneurysm treatment, they underwent external ventriculostomy drain placement for acute hydrocephalus. All patients received enteral nimodipine for the prevention of cerebral ischemia secondary to vasospasm, and they underwent daily surveillance with transcranial Doppler imaging (TCDI). None of the patients received statin therapy. Following the clinical recognition of a neurologic change, all patients underwent urgent brain CT and CTA, as well as CT perfusion (CTP) scans. The severity of vasospasm was characterized by dividing the vessel diameter by the diameter measured on the initial scan [13]. Cerebral ischemia was defined by neurological deterioration (decrease in consciousness and/or the presence of new neurological deficits) not explained by other causes such as infection, hypotension, electrolyte derangements, hydrocephalus, cessation of external ventricular drainage associated with moderate or severe angiographic vasospasm [14,15]. Refractory cerebral ischemia was defined as persistent neurological worsening despite first-line therapy, including induced hypertension and volume expansion while maintaining euvolemia [16,17]. Induced hypertension was characterized by cerebral perfusion pressure of 80-120 mmHg by targeting mean arterial pressure of 90-130 mmHg and maximum systemic blood pressure of 220 mmHg with a combination of volume resuscitation and IV pressor support [18-20]. The euvolemia fluid strategy was pursued, as the positive-fluid-balance strategy in SAH-associated vasospasm has been challenged [20]. After confirmation of vasospasm on digital subtraction angiography (DSA), the patients underwent vasospasm treatment at the discretion of the interventionalist (Table 1).

**Table 1:** Summary of three patients with vasospasm-associated cerebral ischemia in subarachnoid hemorrhage, onset of vasospasm, vasospasm treatment, and the follow-up images with resolution of vasospasm and cerebral ischemia CTA: computed tomographic angiography; CTP: computed tomographic perfusion

Patient	Demographic	Vasospasm onset (day)	Endovascular treatment (day)	Cilostazol treatment (day)	Albumin treatment (day)	Follow-up CTA (day)	Follow-up CTP (day)
1	58 F	9	9	9-14	9-10	11	11
2	67 F	13	13, 15	16-19	16-19	18	18
3	56 F	4	4, 6	6-11	6-7	7	7

All patients were then administered enteral cilostazol (200 mg/day) and IV (25%) albumin (1.25 g per kg over eight hours) treatment for refractory cerebral ischemia [7,8]. Albumin was administered over eight hours to minimize cardiopulmonary adverse side effects. The maximum duration of albumin (seven days) was chosen based on the Albumin in Subarachnoid Hemorrhage (ALISAH) study [7] and the maximum duration of cilostazol (14 days) was chosen based on the meta-analysis performed by Qureshi et al. [8]. The protocol for retrospective data collection and analysis was reviewed and approved by the University of Missouri Institutional Review Board (approval number: 2098328).

Patient 1

**Figure 1:**
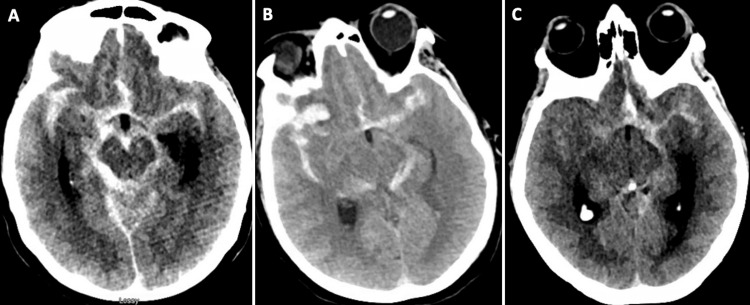
Initial non-contrast CT brain of patient 1 (A), patient 2 (B), and patient 3 (C) demonstrating diffuse subarachnoid hemorrhage

**Figure 2:**
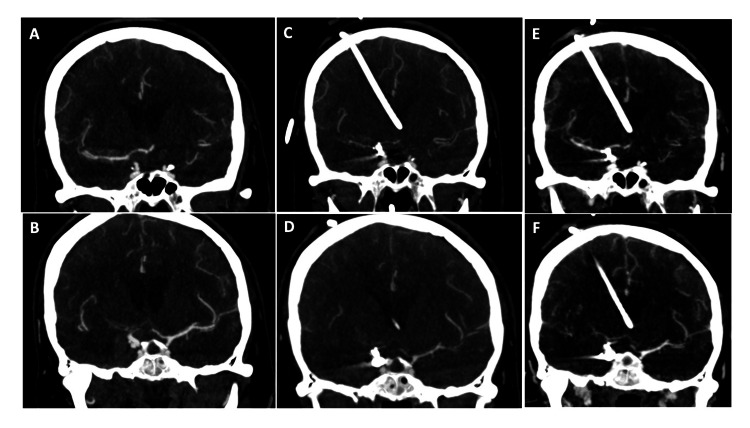
Initial CTA of the right (A) and left (B) anterior hemispheres, CTA demonstrating vasospasm in the right (C) and left (D) anterior hemispheres on day 9, and follow-up CTA demonstrating improvement in vasospasm in the right (E) and left (F) anterior hemispheres two days after initiating albumin and cilostazol therapy in patient 1 CTA: computed tomographic angiography

**Figure 3:**
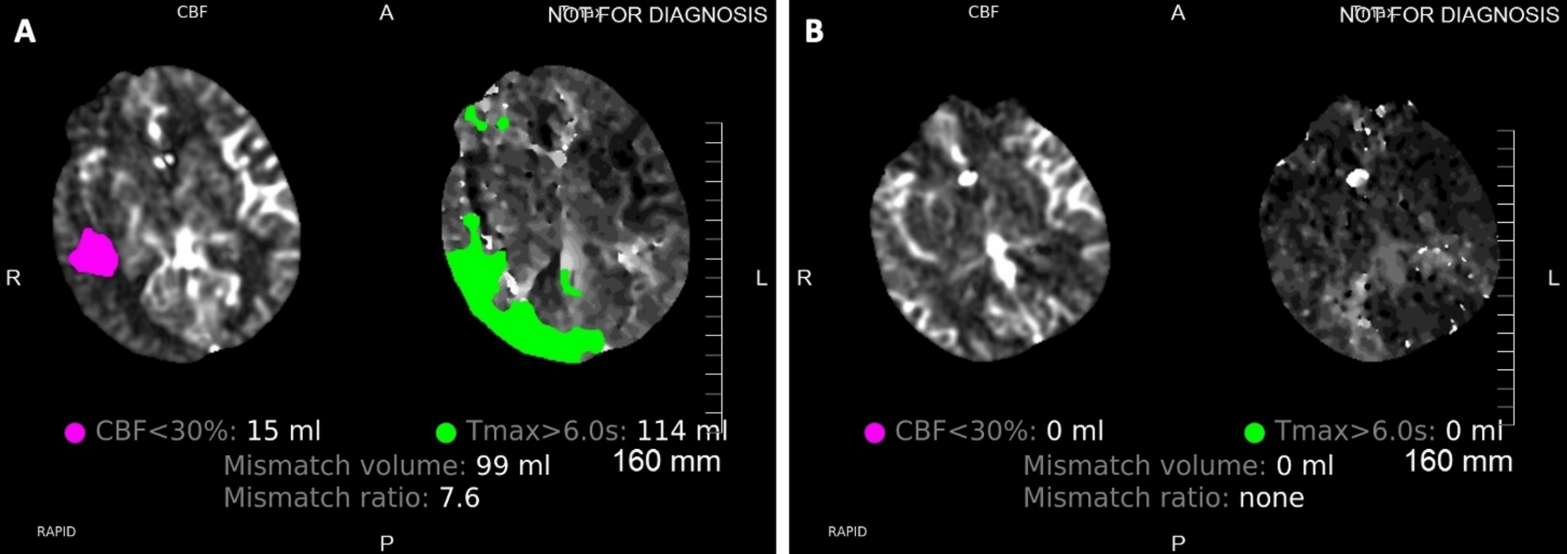
CTP demonstrating perfusion deficits during vasospasm (A) and follow-up CTP with resolved perfusion (B) deficits two days after initiation of cilostazol and albumin treatment in patient 1 CTP: computed tomographic perfusion

A 58-year-old woman presented with moderate-severe headache to an outside facility. A CT brain (Figure 1) demonstrated diffuse SAH involving both Sylvian fissures and the ambient cistern, modified Fischer Score 4, and Hunt and Hess grade 2. A CTA (Figure 2) demonstrated a right posterior communicating artery aneurysm. Her level of consciousness deteriorated on arrival at our facility, progressing to Hunt and Hess grade 4. A digital subtraction angiogram demonstrated a 4 x 6 mm right posterior communicating artery aneurysm. The aneurysm was secured with stent-assisted coil placement, with Raymond Roy Class 1 occlusion (complete occlusion) of the aneurysm. The patient was initiated on daily enteral aspirin 81 mg and IV cangrelor drip at 1 µg/kg/min. On admission on day 9, she displayed left-sided hemiparesis. A CTA demonstrated moderate vasospasm in the right MCA and ACA when compared to the admitting CTA (Figure 2). A CTP (Figure 3) demonstrated perfusion deficits in the right cerebral hemisphere. The digital subtraction angiogram identified severe vasospasm at the right middle cerebral artery, horizontal segment, and right anterior cerebral artery, pre-communicating segment when compared to the initial digital subtraction angiogram. Her vasospasm was treated with a total of 25 mg of intra-arterial nicardipine delivered in the right internal carotid artery. Immediately after the vasospasm treatment, she was transitioned from enteral aspirin and IV cangrelor to enteral aspirin and cilostazol. She was also initiated on IV albumin (Tables 1, 2). A follow-up CTA (Figure 2) on admission day 11 (two days after initiation of cilostazol and albumin) demonstrated improved vessel caliber of the right middle cerebral artery and worse vessel caliber of the right anterior cerebral artery compared to the initial CTA. A follow-up CTP did not identify any perfusion deficits (Figure 3). She received 24 hours of IV albumin and 14 days of enteral cilostazol; she could only tolerate 24 hours of albumin secondary to pulmonary congestion. Serial TCDIs post-treatment demonstrated improved vasospasm in the right middle cerebral artery (Table 2). She required no further vasospasm treatment for the entirety of her hospital stay, and her final CTH did not demonstrate a new area of infarct when compared to the initial CTH.

**Table 2:** Summary of CT angiogram, CT perfusion, and TCDI findings in reference to vasospasm evaluation and treatment in patient 1 MCA: middle cerebral artery; ACA: anterior cerebral artery; Tmax: time-to-maximum of tissue residue function; CBV: cerebral blood volume; MFV: mean flow velocity; TCDI: transcranial Doppler imaging

	Treatment			CT angiogram				CT perfusion		Transcranial Doppler ultrasound	
	Endovascular	Cilostazol (200 mg/day)	Albumin (1.25 gm/kg over eight hours)	Right MCA % reduction	Right ACA % reduction	Left MCA % reduction	Left ACA % reduction	Tmax (cc)	CBV (cc)	MFV right MCA (cm/s)	MFV left MCA (cm/s)
Day 7	-	-	-	-	-	-	-	-	-	32	63
Day 8	-	-	-	-	-	-	-	-	-	145	63
Day 9	Intra-arterial 25 mg Nicardipine	After the vasospasm treatment 200 mg	After the vasospasm treatment 1.25 gm/kg	48	34	11	40	114	15	130	78
Day 10	-	200 mg	1.25 gm/kg	-	-	-	-	-	-	78	92
Day 11	-	200 mg	-	20	50	6	28	0	0	104	117
Day 12	-	200 mg	-	-	-	-	-	-	-	58	100
Day 13	-	200 mg	-	-	-	-	-	-	-	81	N/A
Day 14	-	200 mg	-	-	-	-	-	-	-	133	167

Patient 2

**Figure 4:**
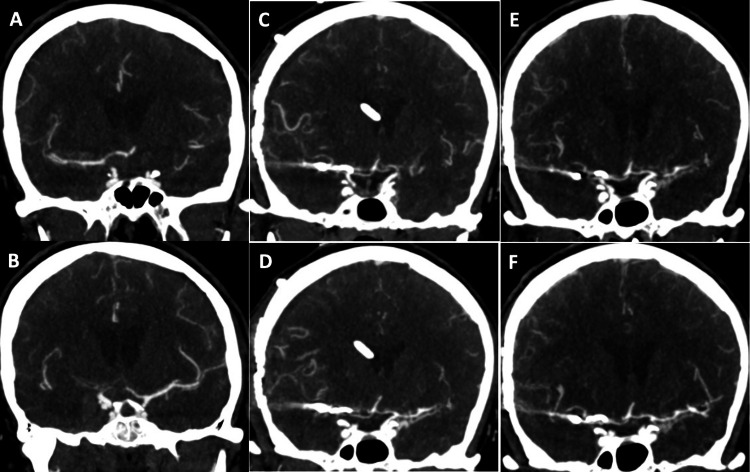
Initial CTA of the right (A) and left (B) anterior hemispheres, CTA demonstrating vasospasm in the right (C) and left (D) anterior hemispheres on day 13, and follow-up CTA demonstrating improvement in vasospasm in the right (E) and left (F) anterior hemispheres two days after initiating albumin and cilostazol therapy in patient 2 CTA: computed tomographic angiography

**Figure 5:**
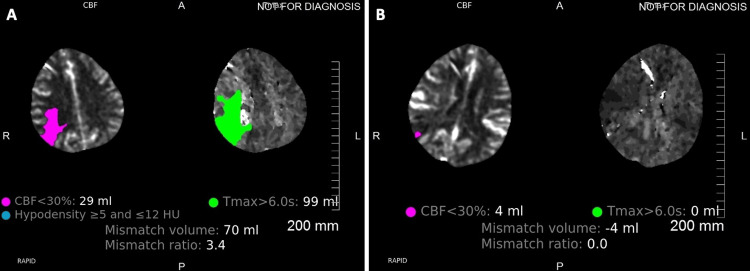
CTP demonstrating perfusion deficits during vasospasm (A) and follow-up CTP with resolved perfusion deficits (B) two days after initiation of cilostazol and albumin treatment in patient 2 CTP: computed tomographic perfusion

A 67-year-old woman presented to our institution in a comatose state with Hunt and Hess grade 4. Her CT brain (Figure 1) demonstrated diffuse SAH involving both Sylvian fissures, ambient, prepontine, and pre-medullary cisterns with a modified Fisher Score of 4. A CTA demonstrated a right middle cerebral artery bifurcation aneurysm (Figure 4). A digital subtraction angiogram demonstrated a 5.5 x 9 mm right middle cerebral artery bifurcation aneurysm. On admission day 1, she underwent surgical clip placement of the aneurysm with Raymond Roy Class 1 occlusion. On admission day 13, she experienced left-sided hemiparesis. A CT brain demonstrated hypodensity in the right frontal lobe consistent with an existing infarct. A CTA (Figure 4) demonstrated severe vasospasm in the right middle cerebral artery, horizontal segment, and moderate vasospasm in the right anterior cerebral artery when compared to the initial CTA. A CTP (Figure 5) was prominent for perfusion deficits in the right cerebral hemisphere, involving the distribution of the middle cerebral artery. A digital subtraction angiogram identified severe vasospasm in the right middle cerebral artery and mild vasospasm in the anterior cerebral artery when compared to the initial digital subtraction angiogram. Her vasospasm was treated with a total of 10 mg of intraarterial verapamil and 30 mg of nicardipine injection at the right internal carotid artery (Table 3). On admission on day 15, she displayed left-sided weakness again. A digital subtraction angiogram identified vasospasm in the right middle cerebral artery and anterior cerebral artery when compared to the initial digital subtraction angiogram. The vasospasm was treated with balloon angioplasty and 10 mg of intraarterial nicardipine injection at the right middle cerebral artery. She was initiated on IV albumin and enteral cilostazol. A follow-up CTA on admission day 18 (two days after initiation of cilostazol and albumin) demonstrated improved vessel caliber of the right middle cerebral artery and anterior cerebral artery when compared to the initial CTA (Figure 4). A follow-up CTP identified minimal perfusion deficits in the right hemisphere (Figure 5). Serial TCDIs post-treatment demonstrated improved vasospasm in the right middle cerebral artery (Table 3). She received IV albumin from admission day 16 to 21, and enteral cilostazol from admission day 16 to 24. She required no further vasospasm treatment for the entirety of her hospital stay, and her final CTH demonstrated a new area of infarct in the right parietal region.

**Table 3:** Summary of CT angiogram, CT perfusion, and TCDI findings in reference to vasospasm evaluation and treatment in patient 2 MCA: middle cerebral artery; ACA: anterior cerebral artery; Tmax: time-to-maximum of tissue residue function; CBV: cerebral blood volume; MFV: mean flow velocity; TCDI: transcranial Doppler imaging

	Treatment			CT angiogram				CT perfusion		Transcranial Doppler ultrasound	
	Endovascular	Cilostazol (200 mg/day)	Albumin (1.25 gm/kg over eight hours)	Right MCA % reduction	Right ACA % reduction	Left MCA % reduction	Left ACA % reduction	Tmax (cc)	CBV (cc)	MFV right MCA (cm/s)	MFV left MCA (cm/s)
Day 12	-	-	-	-	-	-	-	-	-	39	N/A
Day 13	Intra-arterial Verapamil 10 mg and Nicardipine 30 mg	-	-	77	48	31	33	99	29	N/A	98
Day 14	-	-	-	-	-	-	-	-	-	73	N/A
Day 15	Angioplasty of right MCA, and Nicardipine 10 mg	-	-	-	-	-	-	-	-	165	N/A
Day 16	-	200 mg	1.25 gm/kg	-	-	-	-	-	-	96	125
Day 17	-	200 mg	1.25 gm/kg	-	-	-	-	-	-	41	148
Day 18	-	200 mg	1.25 gm/kg	38	22	25	4	0	4	N/A	N/A
Day 19	-	200 mg	1.25 gm/kg	-	-	-	-	-	-	38	N/A

Patient 3

**Figure 6:**
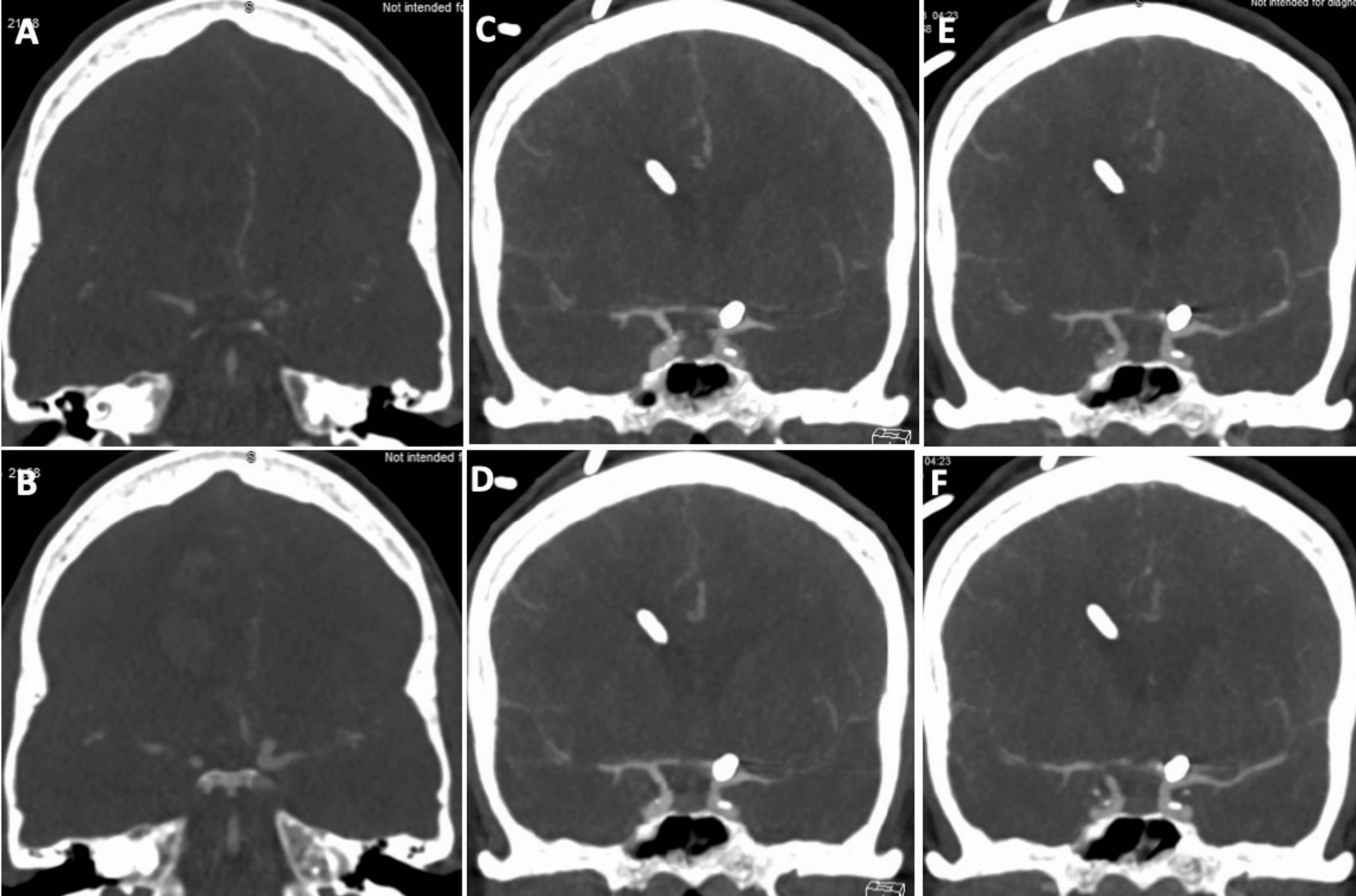
Initial CTA of the right (A) and left (B) anterior hemispheres, CTA demonstrating vasospasm in the right (C) and left (D) anterior hemispheres on day 6, and follow-up CTA demonstrating improvement in vasospasm in the right (E) and left (F) anterior hemispheres two days after initiating albumin and cilostazol therapy in patient 3 CTA: computed tomographic angiography

**Figure 7:**
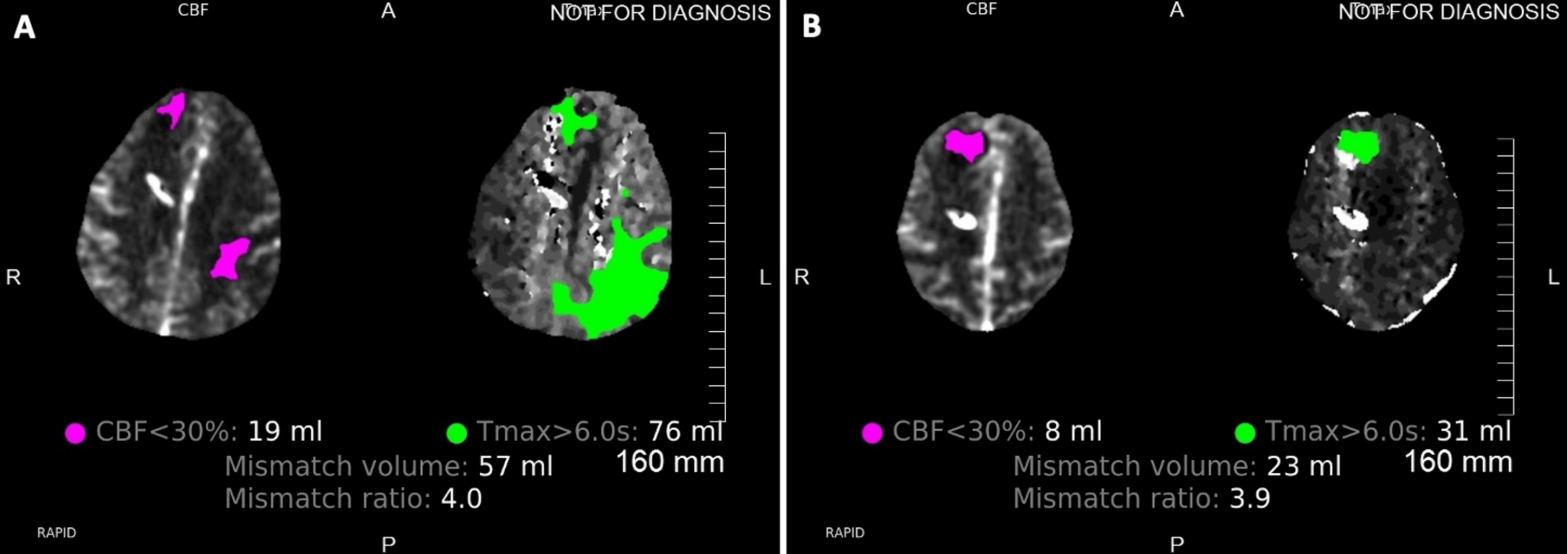
CTP demonstrating perfusion deficits during vasospasm (A) and follow-up CTP with resolved perfusion deficits (B) two days after initiation of cilostazol and albumin treatment in patient 3 CTP: computed tomographic perfusion

A 56-year-old woman presented to our institution in a distressed state with a headache with Hunt and Hess grade 2. Her CT brain (Figure 1) demonstrated a 14 x 30 x 32 mm right frontal intraparenchymal hemorrhage, diffuse SAH involving both Sylvian fissures, ambient, prepontine, and pre-medullary cisterns with a modified Fisher Score of 4. A CTA (Figure 6) demonstrated a left internal carotid artery terminus segment aneurysm. On admission day 1, a digital subtraction angiogram demonstrated a 3.9 x 5.6 mm left internal carotid artery terminus aneurysm. She underwent balloon-assisted coiling of the aneurysm with Raymond Roy Class 2 occlusion. On admission on day 4, she underwent a follow-up digital subtraction angiogram, which demonstrated moderate vasospasm of the right middle cerebral artery, mild vasospasm of the right anterior cerebral artery, and severe vasospasm of the left anterior and middle cerebral arteries, treated with 5 mg of intraarterial Verapamil at the right and left internal carotid artery, respectively. On admission day 6, she displayed prosopagnosia and aphasia. A CTA (Figure 6) demonstrated mild vasospasm in the right middle cerebral artery, mild vasospasm in the left anterior cerebral artery, and severe vasospasm in the left middle cerebral artery when compared to the initial CTA. A CTP (Figure 7) was prominent for perfusion deficits in both cerebral hemispheres, predominantly in the distribution of the left middle cerebral artery. A digital subtraction angiogram identified moderate vasospasm in the right anterior cerebral artery, moderate vasospasm in the left anterior cerebral artery, and severe vasospasm in the left middle cerebral artery when compared to the initial digital subtraction angiogram. Her vasospasm was treated with 25 mg of intra-arterial nicardipine injection at the left internal carotid artery and 10 mg of nicardipine injection at the right internal carotid artery. She was initiated on IV albumin and enteral cilostazol. A follow-up CTA (Figure 6) on admission day 7 (two days after initiation of cilostazol and albumin) demonstrated improved vessel caliber of the right anterior cerebral, right middle cerebral artery, left anterior cerebral artery, and left middle cerebral artery when compared to the initial CTA. Figure 6 denotes a vessel diameter larger than the diameter measured on the initial scan, indicating some degree of vasospasm on the initial scan. A follow-up CTP identified minimal perfusion deficits in the right hemisphere and improved perfusion deficits in the left cerebral hemisphere (Figure 7). Serial TCDIs post-treatment demonstrated improved vasospasm in the left middle cerebral artery (Table 4). She received IV albumin from admission days 5 to 6 and enteral cilostazol from admission days 5 to 18; she could only tolerate two days of albumin due to pulmonary congestion. She required no further vasospasm treatment for the entirety of her hospital stay, and her final CTH did not demonstrate any new area of infarct.

**Table 4:** Summary of CT angiogram, CT perfusion, and TCDI findings in reference to vasospasm evaluation and treatment in patient 3 MCA: middle cerebral artery; ACA: anterior cerebral artery; Tmax: time-to-maximum of tissue residue function; CBV: cerebral blood volume; MFV: mean flow velocity; TCDI: transcranial Doppler imaging The percentage reduction (i.e., the severity of vasospasm) is calculated by dividing the vessel diameter by the diameter measured on the initial scan; * denotes the vessel diameter larger than the diameter measured on the initial scan, which may indicate some degree of vasospasm on the initial scan.

	Treatment			CT angiogram				CT perfusion		Transcranial Doppler ultrasound	
	Endovascular	Cilostazol (200 mg/day)	Albumin (1.25 gm/kg over eight hours)	Right MCA % reduction	Right ACA % reduction	Left MCA % reduction	Left ACA % reduction	Tmax (cc)	CBV (cc)	MFV right MCA (cm/s)	MFV left MCA (cm/s)
Day 4	Intra-arterial Verapamil 5 mg at both internal carotid arteries	-	-	-	-	-	-	-	-	115	124
Day 5	-	-	-	-	-	-	-	-	-	113	135
Day 6	Intra-arterial Nicardipine 10 mg at right and 25 mg at left internal carotid artery	After the intra-arterial vasospasm treatment 200 mg	After the intra-arterial vasospasm treatment 1.25 gm/kg	23	36*	22	53	76	19	148	110
Day 7	-	200 mg	1.25 gm/kg	62*	73*	67*	20	31	8	52	85
Day 8	-	200 mg	-	-	-	-	-	-	-	N/A	N/A
Day 9	-	200 mg	-	-	-	-	-	-	-	124	150
Day 10	-	200 mg	-	-	-	-	-	-	-	161	177
Day 11	-	200 mg	-	-	-	-	-	-	-	59	101

In patient 1, the middle cerebral artery percent luminal reduction improved from 48% to 20% on day 11 (two days post albumin and cilostazol treatment) and improved further to 1% on day 13 (four days post albumin and cilostazol treatment). The anterior cerebral artery percent luminal reduction increased from 34% to 50% on day 11 and decreased to 1% on day 13 when compared to the initial CTA (Figures 2 and Table 2). The volume of cerebral ischemia and ischemic core improved from 114 cc and 15 cc to 0 cc on days 11 and 13 (Figure 3). Similarly, the mean flow velocity (MFV) of MCA on TCDI improved from 130 cm/s to 104 cm/s and 81 cm/s on days 11 and 13, respectively (Table 2).

In patient 2, the middle cerebral artery percent luminal reduction improved from 77% to 38%, and the anterior cerebral artery percent luminal reduction improved from 48% to 22% on day 18 when compared to the initial CTA (Figure 4 and Table 3). The volume of cerebral ischemia and ischemic core improved from 99 cc and 29 cc to 3 cc and 6 cc, respectively, on day 18 (Figure 5). The MFV of the middle cerebral artery on TCDI was not measurable on the day of vasospasm; however, it progressively improved over the next few days. (Table 3).

In patient 3, the right anterior cerebral artery percent luminal reduction improved from 23% to 62%, the right middle cerebral artery from 36% to 73%, the left anterior cerebral artery from 22% to 67%, and the left MCA percent luminal reduction improved from 53% to 20% on day 7 (two days post albumin and cilostazol treatment), when compared to the initial CTA (Figure 6 and Table 4). The volume of cerebral ischemia and ischemic core improved from 76 cc and 19 cc to 31 cc and 8 cc, respectively, on day 7 (Figure 7). The MFV of the MCA on TCDI progressively improved over the next few days (Table 4).

## Discussion

We observed sustained resolution of refractory cerebral ischemia in three aSAH patients based on findings on CTP perfusion and TCDI and resolution/non-progression of clinical deficits. The resolution of vasospasm on CTA and TCDI and perfusion deficits on CTP suggested that the combination of albumin and cilostazol may act on both medium-sized and small arteries and arterioles. The treatment of medium-sized arterial vasospasm with endovascular means, including intra-arterial vasodilators and balloon angioplasty alone, does not explain the complete resolution of perfusion on post-endovascular treatment CTPs [21]. Furthermore, in the patients who received intra-arterial vasodilators, the sustained amelioration would be inconsistent with the short-lived actions of intra-arterial vasodilators [22,23]. It is possible that cerebral ischemia resolved spontaneously as part of the natural history of cerebral ischemia and vasospasm, but the time course of resolution correlated strongly with the initiation of enteral cilostazol and IV albumin [24]. The effect of both agents is complementary since IV albumin improves flow through microcirculation, and enteral cilostazol reduces proliferative changes in medium-sized and small arteries and prevents platelet aggregation [8,25-29]. In aSAH patients who are on multiple treatments, the exact role of the combination of cilostazol and IV albumin in the amelioration of cerebral ischemia needs to be considered. Our findings highlight the therapeutic potential of enteral cilostazol and high-dose IV albumin combination for refractory cerebral ischemia associated with aSAH by potentially influencing multiple pathways.

We observed that two patients could only tolerate two doses of IV albumin, and further doses were not used to avoid exacerbation of fluid overload. In the albumin dose-finding trial (ALISAH), Suarez et al. [7] demonstrated that while all the aSAH patients in the 0.625 g/kg/day tier tolerated the treatment, one patient receiving 1.25 g/kg/day and two patients receiving 1.875 g/kg/day were not able to tolerate the treatment due to pulmonary edema [7]. The difference in tolerance of IV albumin in our patients and ALISAH is probably attributed to existing fluid overload in our patients due to induced hypervolemia with or without induced hypertension for the treatment of cerebral ischemia. To mitigate hypervolemia, dosing over a longer duration (i.e., over eight hours compared to the three hours in the ALISAH study) used in our patients or monitoring of central venous pressure to maintain between 5 mmHg and 8 mmHg can be considered [7]. The concomitant use of albumin with IV diuretics is another consideration used in the ALIAS trial [6,25]. Even though the pilot study of the ALIAS trial demonstrated a higher likelihood of good outcomes with higher doses of albumin, the formal trial terminated due to safety reasons, predominantly from cardiopulmonary complications [6]. However, the authors demonstrated that pulmonary edema/congestive heart failure was remedied through fluid management and diuretic use [30]. Therefore, concomitant use of albumin with diuretics may remedy the fluid overload in aSAH patients.

Concomitant use of cilostazol may increase the fluid overload with IV albumin. Although the use of cilostazol in patients with existing heart failure is contraindicated, it has not been shown to directly cause congestive heart failure [31,32]. We did not observe any premature discontinuation of cilostazol due to tachycardia as reported in previous aSAH studies [33]. We also did not observe any bleeding complications, particularly intracranial bleeding. Cilostazol was discontinued after the highest risk period for vasospasm in all patients; therefore, the patients were able to undergo intraventricular drain placement without increased risk of intracranial bleeding.

## Conclusions

A combination of enteral cilostazol and IV high-dose albumin was associated with amelioration of angiographic vasospasm and perfusion deficits in aSAH patients with severe refractory cerebral ischemia, presumably via multiple pathways that involve improved flow through microcirculation and reduction of proliferative changes in medium-sized and small arteries and prevention of platelet aggregation. While various treatments, including intra-arterial vasodilator delivery and intracranial angioplasty, may introduce confounding factors, sustained improvement in vasospasm is not solely explained by the short half-life of vasodilators. A major limitation of our results is the small number of patients (n = 3) in our case series; the role of the combination of enteral cilostazol and IV albumin in the amelioration of cerebral ischemia needs to be explored in a larger case series and possibly in a randomized controlled trial.

## References

[1] Gempeler A, Gaviria L, Ortiz A (2023). Effect of an albumin infusion treatment protocol on delayed cerebral ischemia and relevant outcomes in patients with subarachnoid hemorrhage. Neurocrit Care.

[2] Crowley RW, Medel R, Dumont AS (2011). Angiographic vasospasm is strongly correlated with cerebral infarction after subarachnoid hemorrhage. Stroke.

[3] Powers WJ, Rabinstein AA, Ackerson T (2019). Guidelines for the early management of patients with acute ischemic stroke: 2019 update to the 2018 guidelines for the early management of acute ischemic stroke: a guideline for healthcare professionals from the American Heart Association/American Stroke Association. Stroke.

[4] Hoh BL, Ko NU, Amin-Hanjani S (2023). 2023 Guideline for the management of patients with aneurysmal subarachnoid hemorrhage: a guideline from the American Heart Association/American Stroke Association. Stroke.

[5] Chan AY, Choi EH, Yuki I (2021). Cerebral vasospasm after subarachnoid hemorrhage: developing treatments. Brain Hemorrhages.

[6] Huang Y, Xiao Z (2021). Albumin therapy for acute ischemic stroke: a meta-analysis. Neurol Sci.

[7] Suarez JI, Martin RH, Calvillo E (2012). The Albumin in Subarachnoid Hemorrhage (ALISAH) multicenter pilot clinical trial: safety and neurologic outcomes. Stroke.

[8] Qureshi AI, Ishfaq A, Ishfaq MF (2018). Therapeutic benefit of cilostazol in patients with aneurysmal subarachnoid hemorrhage: a meta-analysis of randomized and nonrandomized studies. J Vasc Interv Neurol.

[9] Merlot AM, Kalinowski DS, Richardson DR (2014). Unraveling the mysteries of serum albumin - more than just a serum protein. Front Physiol.

[10] Wang L, Li M, Xie Y, Xu L, Ye R, Liu X (2017). Preclinical efficacy of human albumin in subarachnoid hemorrhage. Neuroscience.

[11] Hunt WE, Hess RM (1968). Surgical risk as related to time of intervention in the repair of intracranial aneurysms. J Neurosurg.

[12] Jaja BN, Saposnik G, Lingsma HF (2018). Development and validation of outcome prediction models for aneurysmal subarachnoid haemorrhage: the SAHIT multinational cohort study. BMJ.

[13] Dankbaar JW, Rijsdijk M, van der Schaaf IC, Velthuis BK, Wermer MJH, Rinkel GJE (2009). Relationship between vasospasm, cerebral perfusion, and delayed cerebral ischemia after aneurysmal subarachnoid hemorrhage. Neuroradiology.

[14] Vergouwen MD, Vermeulen M, van Gijn J (2010). Definition of delayed cerebral ischemia after aneurysmal subarachnoid hemorrhage as an outcome event in clinical trials and observational studies: proposal of a multidisciplinary research group. Stroke.

[15] Neumann A, Küchler J, Ditz C, Krajewski K, Leppert J, Schramm P, Schacht H (2021). Non-compliant and compliant balloons for endovascular rescue therapy of cerebral vasospasm after spontaneous subarachnoid haemorrhage: experiences of a single-centre institution with radiological follow-up of the treated vessel segments. Stroke Vasc Neurol.

[16] Al-Mufti F, Amuluru K, Damodara N (2018). Novel management strategies for medically-refractory vasospasm following aneurysmal subarachnoid hemorrhage. J Neurol Sci.

[17] Abulhasan YB, Ortiz Jimenez J, Teitelbaum J, Simoneau G, Angle MR (2021). Milrinone for refractory cerebral vasospasm with delayed cerebral ischemia. J Neurosurg.

[18] Azari Jafari A, Mirmoeeni S, Johnson WC (2023). The effect of induced hypertension in aneurysmal subarachnoid hemorrhage: a narrative review. Curr J Neurol.

[19] Gathier CS, van den Bergh WM, van der Jagt M (2018). Induced hypertension for delayed cerebral ischemia after aneurysmal subarachnoid hemorrhage: a randomized clinical trial. Stroke.

[20] Gál J, Fülesdi B, Varga D (2020). Assessment of two prophylactic fluid strategies in aneurysmal subarachnoid hemorrhage: a randomized trial. J Int Med Res.

[21] Omoto K, Nakagawa I, Nishimura F, Yamada S, Motoyama Y, Nakase H (2020). Computed tomography perfusion imaging after aneurysmal subarachnoid hemorrhage can detect cerebral vasospasm and predict delayed cerebral ischemia after endovascular treatment. Surg Neurol Int.

[22] Lim J, Cho YD, Kwon HJ, Byoun SH, Koh HS, Park B, Choi SW (2021). Duration of vasodilatory action after intra-arterial infusions of calcium channel blockers in animal model of cerebral vasospasm. Neurocrit Care.

[23] Connolly ES, Rabinstein AA, Carhuapoma JR (2012). Guidelines for the management of aneurysmal subarachnoid hemorrhage. Stroke.

[24] Dodd WS, Laurent D, Dumont AS (2021). Pathophysiology of delayed cerebral ischemia after subarachnoid hemorrhage: a review. J Am Heart Assoc.

[25] Senbokuya N, Kinouchi H, Kanemaru K (2013). Effects of cilostazol on cerebral vasospasm after aneurysmal subarachnoid hemorrhage: a multicenter prospective, randomized, open-label blinded end point trial. J Neurosurg.

[26] Nishimura N, Schaffer CB, Friedman B, Lyden PD, Kleinfeld D (2007). Penetrating arterioles are a bottleneck in the perfusion of neocortex. Proc Natl Acad Sci U S A.

[27] Herz DA, Baez S, Shulman K (1975). Pial microcirculation in subarachnoid hemorrhage. Stroke.

[28] Pennings FA, Bouma GJ, Ince C (2004). Direct observation of the human cerebral microcirculation during aneurysm surgery reveals increased arteriolar contractility. Stroke.

[29] Defazio RA, Zhao W, Deng X, Obenaus A, Ginsberg MD (2012). Albumin therapy enhances collateral perfusion after laser-induced middle cerebral artery branch occlusion: a laser speckle contrast flow study. J Cereb Blood Flow Metab.

[30] Martin RH, Yeatts SD, Hill MD, Moy CS, Ginsberg MD, Palesch YY (2016). Alias (albumin in acute ischemic stroke) trials: analysis of the combined data from parts 1 and 2. Stroke.

[31] Wu CK, Lin JW, Wu LC, Chang CH (2018). Risk of heart failure hospitalization associated with cilostazol in diabetes: a nationwide case-crossover study. Front Pharmacol.

[32] Lee YC, Lin JW, Wu LC, Chang CH (2016). Risk of hospitalization for heart failure associated with cilostazol in patients with diabetes mellitus: a nationwide case-crossover study. J Card Fail.

[33] Gamssari F, Mahmood H, Ho JS (2002). Rapid ventricular tachycardias associated with cilostazol use. Tex Heart Inst J.

